# Semi-field evaluation of freestanding transfluthrin passive emanators and the BG sentinel trap as a “push-pull control strategy” against *Aedes aegypti* mosquitoes

**DOI:** 10.1186/s13071-020-04263-3

**Published:** 2020-07-31

**Authors:** Mgeni M. Tambwe, Sarah J. Moore, Hassan Chilumba, Johnson K. Swai, Jason D. Moore, Caleb Stica, Adam Saddler

**Affiliations:** 1 Environmental Health and Ecological Sciences, Ifakara Health Institute, P.O. Box 74, Bagamoyo, Tanzania; 2 Swiss Tropical & Public Health Institute, Socinstrasse 57, 4051 Basel, Switzerland; 3 University of Basel, Petersplatz 1, 4001 Basel, Switzerland

**Keywords:** Spatial repellent, Odor-baited trap, FTPE, Push-pull, BG-sentinel trap, Transfluthrin, *Aedes aegypti*

## Abstract

**Background:**

Spatial repellents that drive mosquitoes away from treated areas, and odour-baited traps, that attract and kill mosquitoes, can be combined and work synergistically in a push-pull system. Push-pull systems have been shown to reduce house entry and outdoor biting rates of malaria vectors and so have the potential to control other outdoor biting mosquitoes such as *Aedes aegypti* that transmit arboviral diseases. In this study, semi-field experiments were conducted to evaluate whether a push-pull system could be used to reduce bites from *Aedes* mosquitoes.

**Methods:**

The push and pull under investigation consisted of two freestanding transfluthrin passive emanators (FTPE) and a BG sentinel trap (BGS) respectively. The FTPE contained hessian strips treated with 5.25 g of transfluthrin active ingredient. The efficacies of FTPE and BGS alone and in combination were evaluated by human landing catch in a large semi-field system in Tanzania. We also investigated the protection of FTPE over six months. The data were analyzed using generalized linear mixed models with binomial distribution.

**Results:**

Two FTPE had a protective efficacy (PE) of 61.2% (95% confidence interval (CI): 52.2–69.9%) against the human landing of *Ae. aegypti*. The BGS did not significantly reduce mosquito landings; the PE was 2.1% (95% CI: −2.9–7.2%). The push-pull provided a PE of 64.5% (95% CI: 59.1–69.9%). However, there was no significant difference in the PE between the push-pull and the two FTPE against *Ae. aegypti* (*P* = 0.30). The FTPE offered significant protection against *Ae. aegypti* at month three, with a PE of 46.4% (95% CI: 41.1–51.8%), but not at six months with a PE of 2.2% (95% CI: −9.0–14.0%).

**Conclusions:**

The PE of the FTPE and the full push-pull are similar, indicative that bite prevention is primarily due to the activity of the FTPE. While these results are encouraging for the FTPE, further work is needed for a push-pull system to be recommended for *Ae. aegypti* control. The three-month protection against *Ae. aegypti* bites suggests that FTPE would be a useful additional control tool during dengue outbreaks, that does not require regular user compliance.
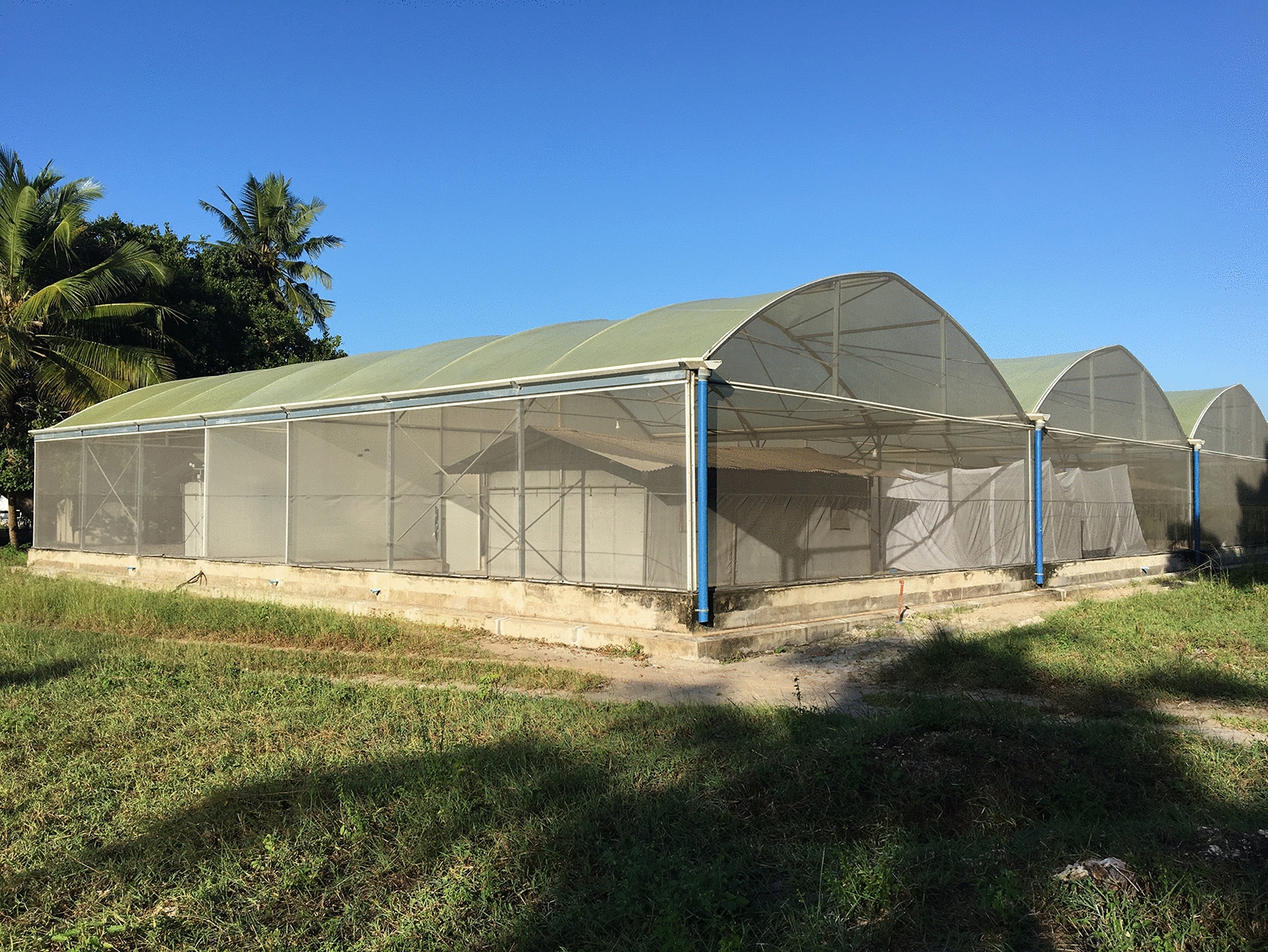

## Background

The *Aedes aegypti* mosquito is the primary vector of many arboviral diseases of public health importance, including dengue, yellow fever, Zika, and chikungunya [1–3]. The risk of contracting an arboviral disease is increasing as the world becomes more urbanized, because *Ae. aegypti* thrive in urban settings [4]. Dengue vector control is centered on larval source management, treatment of resting surfaces with insecticides and with space spraying as a response to disease outbreaks [5]. However, insecticides used for both space spraying and larviciding are short lasting and require a high frequency of reapplication to achieve sustained vector control. Larval habitat reduction is more sustainable but is not always practically or economically feasible in dengue-endemic countries.

Personal protection measures are also recommended during disease outbreaks through the use of appropriate clothing or topical repellents [6]. Topical repellents such as DEET (N, N-diethyl-m-toluamide) have demonstrated efficacy in reducing mosquito bites [7], and are also recommended for arbovirus prevention among military personnel and travelers [8]. However, there have been no studies to demonstrate their efficacy in reducing arboviral disease transmission. Topical repellents require frequent reapplication, which inevitably results in poor user compliance, and consequently coverage levels that are insufficient to interrupt disease transmission [9]. Each of the current control tools for *Aedes* has clear limitations and therefore, the development of complementary control tools to help fill these gaps is needed.

Spatial repellents [10, 11] and odor-baited traps [12, 13] have been suggested for the control of *Aedes* mosquitoes. Spatial repellents provide a bite-free space using repellent chemicals that passively evaporate at room temperature (emanators) or that are actively dispersed through heating coils, mats or vaporizers [14]. By removing the need for individual application, higher coverage levels may be possible with spatial repellents compared to topical repellents. Furthermore, spatial repellents may last for days, even months, after one application reducing the hassle of re-application and potentially increasing the protection even further through high effective coverage. Hessian strips treated with transfluthrin used as passive emanators have been shown to reduce human landing rates by > 90% for *Culex* and *Anopheles* mosquitoes in both semi-field and field experiments for up to six months [11, 15].

Odor baited traps, have been used extensively for mosquito monitoring and have recently been found to have public health benefits by reducing the population density of malaria and dengue vectors when deployed at a large scale in sufficient numbers to ultimately decrease disease transmission [13, 16, 17]. It has been shown that both spatial repellents and odor-baited traps used individually can be effective for the control of *Ae. aegypti*. These tools work by providing personal protection through reducing human-vector contact and also providing community protection by reducing the mosquito population size.

The push-pull control strategy originated from studies of agricultural pests showing that the practice of repelling “pushing” insects from one area and attracting “pulling” them to another, increases crop production [18]. The same strategy may be applied to control disease-transmitting mosquitoes of public health importance using spatial repellents and odor baited traps [19] and mathematical models have predicted that this control strategy may reduce entomological inoculation rate (EIR) by 20-fold for indoor-biting malaria-transmitting mosquitoes [19]. While push-pull control tools have also been tested against *Aedes*, successful laboratory results did not transfer to semi-field settings [14] with the researchers hypothesizing that the spatial repellent chemicals did not reach effective concentrations. In this study we investigate a new push-pull combination for *Aedes* using technologies that have individually proven successful under semi-field and field conditions. For the push component, a transfluthrin-treated hessian strip [15] was adapted to make a freestanding transfluthrin passive emanator (FTPE). The widely studied BGS was selected as the pull component of the system. Here, we investigate the efficacy of push and pull individually and then in a combination as push-pull to see if the combination provided better efficacy measured as a reduction in mosquito landings than either of the individual components. We also measured the duration of protective efficacy of the FTPE over a six-month period.

## Methods

### Study design

This study investigated the efficacy of the FTPE and BGS in a push-pull system to reduce human-landing rates compared to the control (no intervention). We also determined if the combination of FTPE and BGS was better than either FTPE or BGS alone whereby the following treatment arms were compared: (i) two FTPE *vs* negative control; (ii) BGS trap *vs* negative control; and (iii) the combination of FTPE and BGS *vs* negative control. The study design was a randomised block design over 16 days per treatment arm. Each intervention and its control were assigned to one of two separate compartments in the semi-field system (SFS) for a block of 4 days, after which the treatment and its control were switched between compartments. Preliminary experiments showed that removing FTPE immediately after experiment and airing the compartment for 20 h was enough to prevent any residual effect of the transfluthrin. In each block of 4 days, 4 volunteers rotated daily between chambers.

### Study site

The experiment was conducted in the SFS located in Bagamoyo, Tanzania, between January 2018 and December 2018. The SFS measures  21 × 29 × 4.5 m with two compartments (21 × 9 m), separated by a corridor. A heavy-duty polyethene wall separates these compartments preventing air movement between the chambers and reducing any chance of cross-contamination when working with spatial repellents or other aerosols. The SFS allows for controlled experiments with set densities of disease-free mosquitoes to be conducted under field-like climatic conditions throughout the year [20].

### Mosquitoes

Laboratory reared, pyrethroid susceptible *Ae. aegypti* (Bagamoyo strain) were used. Susceptibility bioassays performed prior the implementation of the experiment following the World Health Organization (WHO) guidelines [21] showed that mortality of these mosquitoes was > 99% after exposure to all the pyrethroid insecticides tested (deltamethrin (0.03%), permethrin (0.25%) and alpha-cypermethrin (0.03%). These mosquitoes were colonized from Bagamoyo in December 2015. Larvae were fed on Tetramin^**®**^ fish food and adult mosquitoes on 10% sucrose *ad libitum*, and cow blood meals (heparinized) were given to adult females for egg production using a membrane-feeding assay. The colony was maintained at 27 ± 5 °C and 50–99% relative humidity.

For this experiment, 3–8 day-old female mosquitoes, previously unfed with blood were used. These mosquitoes were sugar-starved for 12 h before the start of experiments. Female mosquitoes that were responsive to human odour were selected from three different rearing cages and transferred to small releasing cages. Selection from cages was done by placing a hand close to the cage and choosing only aggressive host-seeking mosquitoes.

### Preparation of the freestanding transfluthrin passive emanator (FTPE)

**Fig. 1:**
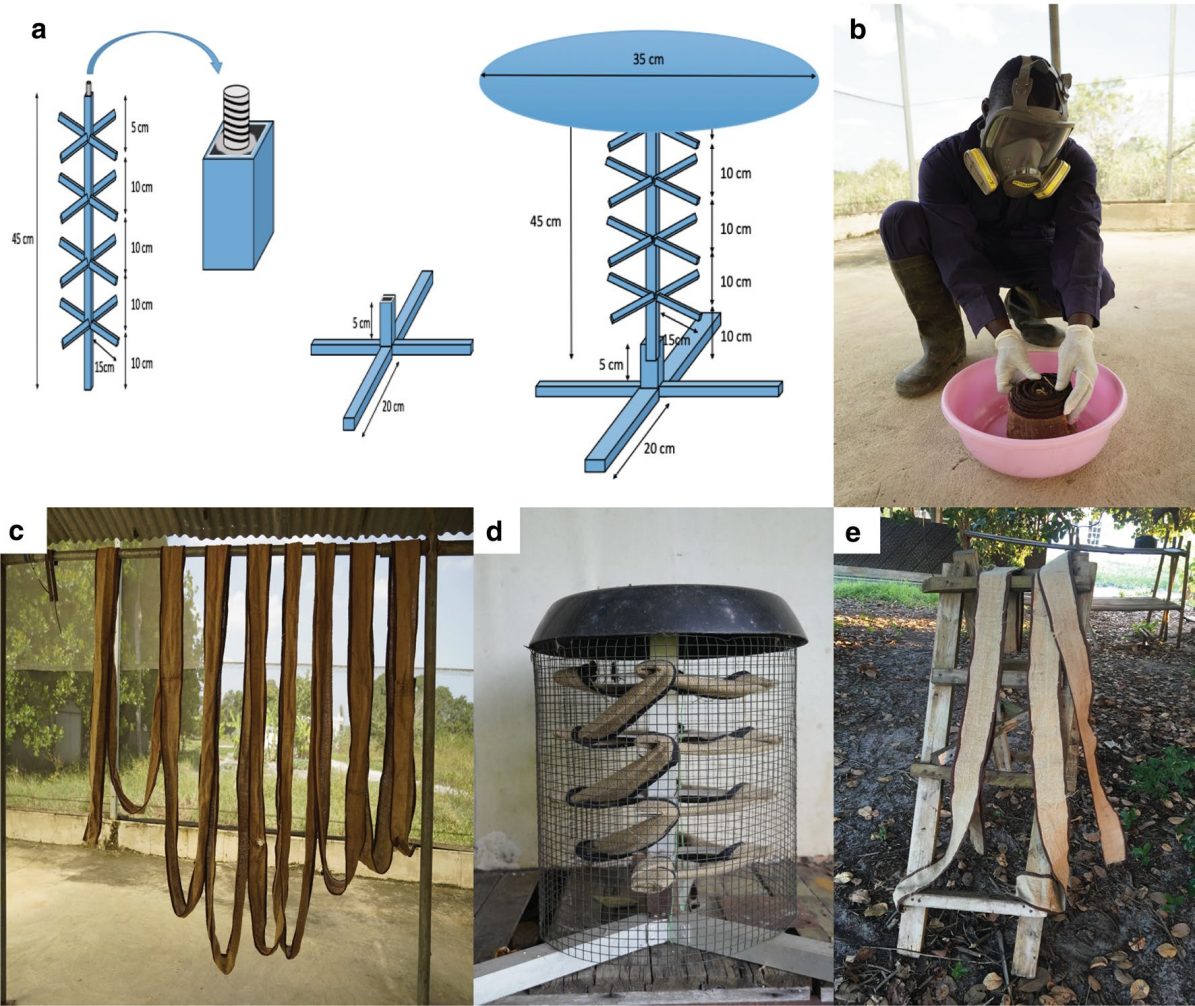
Preparation of the freestanding emanator (FTPE) “push”. **a** Design of the freestanding emanator. The device measures 50 cm in height and 40 cm in diameter. It consists of three parts; the top cover, the central square pipe and a base. The central pipe rests on the base that supports the device. The pipe is divided into four portions 10 cm apart where small branches of an aluminium flat bar 15 cm long are attached. **b**, **c** Transfluthrin impregnation and drying of the hessian strips under the shade. **d** The FTPE (the hessian strip enclosed with the wire mesh). **e** The transfluthrin-treated hessian strips placed under the shade between the experiment for “field aging” for the duration of efficacy experiment

We designed a device that can easily be placed anywhere in the peri-domestic space (Fig. 1a–e). The emanator passively releases transfluthrin vapors into the surrounding area through evaporation. The device is a stool-like structure that supports hessian strips (made from plants of the species *Corchorus olitorius* or *C. capsularis* also called jute, burlap or gunny sacks), treated with the emulsifiable concentrate (EC) transfluthrin active ingredient (Bayothrin EC; Bayer AG Monheim am Rhein, Germany) as the push. The hessian fabrics were chosen as they have been shown to retain transfluthrin for up to 6 months due to their high cellulose content [11, 15]. The hessian fabric was locally bought, washed with OMO^**®**^ detergent powder (Unilever Kenya Limited, Nairobi, Kenya) and dried under direct sunlight. The fabrics were cut into strips with a surface area of 0.5 m^2^ (10 cm × 5 m) and treated with 5.25 g of emulsified concentrate transfluthrin and left to dry under the shade in the SFS (Fig. 1b, c) to prevent photolysis of transfluthrin. The strips were then wound around a pole into a spiral and sealed with outer wire mesh to prevent access to the treated hessian ribbon by children or animals (Fig. 1d). Two FTPE with a total of 10.50 g (5.25 g each) of transfluthrin were used per experiment.

### BG sentinel trap

The BGS (Biogents AG, Regensburg, Germany) has been widely used as the standard trap for collection of adult *Aedes* mosquitoes [22, 23]. The BGS was used with a Biogents-Lure (BGL) and carbon dioxide as a pull. The BGL is a synthetic lure consisting of lactic acid, caproic acid and ammonium bicarbonate dispensed *via* granules [23]. It is effective for 5 months; however, for the purpose of this experiment a new lure was used for each experimental round of sixteen days. Carbon dioxide was released from a pressurized cylinder at the rate of 500 ml/min, using an acrylic gas flow meter (Hangzhou Darhor, Technology Co. Limited, Zhejiang, China).

### Procedure to determine the protective efficacy of the FTPE and odor baited trap

**Fig. 2:**
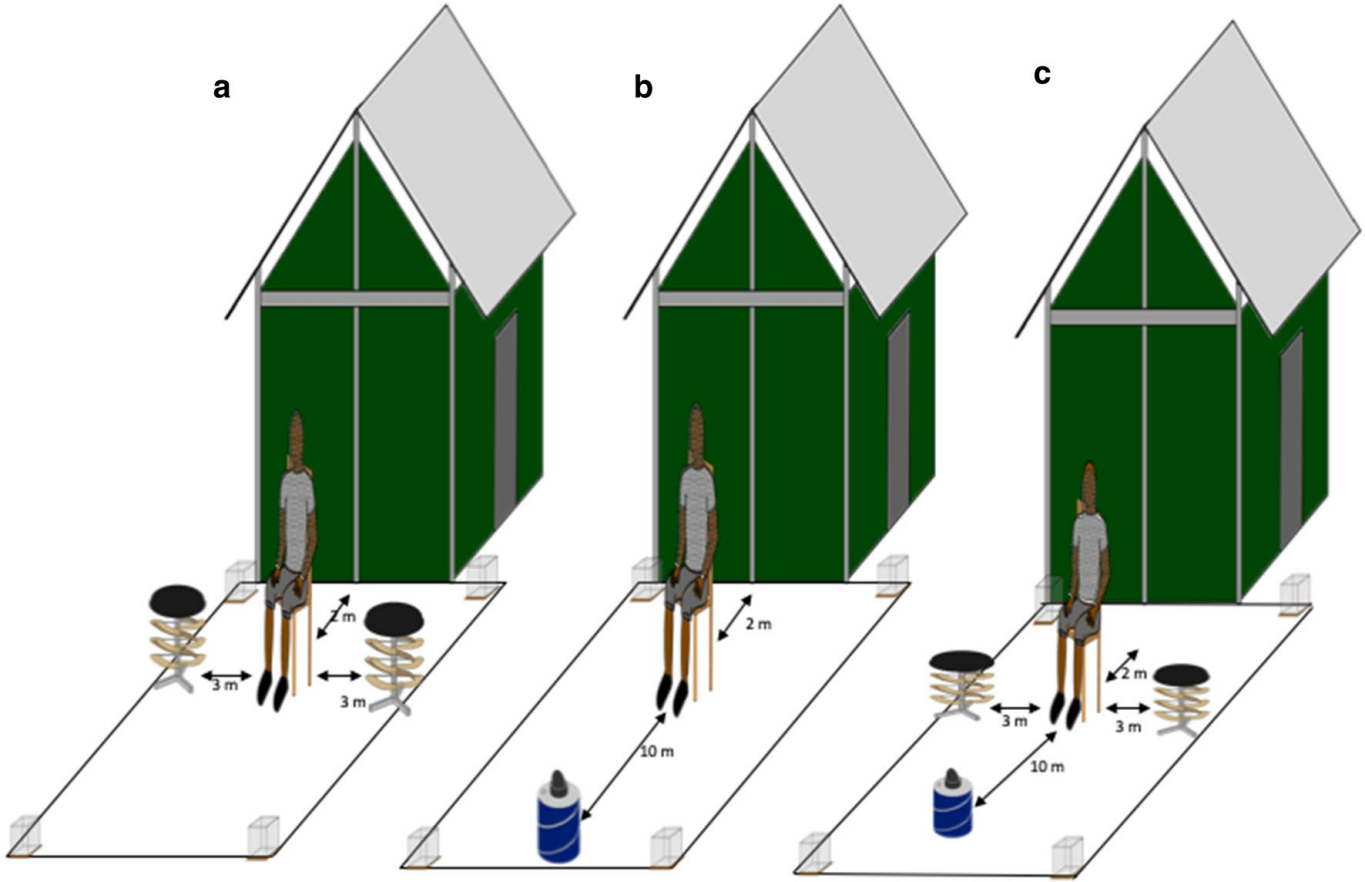
Schematic representation for the experiment in the SFS. **a** The arrangement of push intervention. **b** The BGS positioned 10 m away from the human volunteer during the pull alone evaluation. **c** The positions of interventions during the push-pull evaluation. In each setup, a human volunteer preforming HLC sat 2 m away from the experimental hut and if push was involved, two FTPE were positioned 3 m on each side of the HLC volunteer. Small boxes at the corner represent the releasing cages positioned where mosquitoes were released

To simulate the peridomestic setting, human landing catches (HLC) were performed with a volunteer sitting 2 m from an experimental hut inside the SFS (Fig. 2a–c). For the “push” alone evaluation, two FTPE were positioned 6 m apart with the human volunteer sitting in between them conducting the HLC (Fig. 2a). During the “pull” alone evaluation, the BG-sentinel was placed 10 m away from the HLC (Fig. 2b) as this exceeds the maximum distance over which mosquitoes select between hosts so that the action of the trap could be measured independently of the effect of the human collector [24]. For the push-pull evaluation, both FTPE and the BGS were used and positioned as described above in the push and pull setups (Fig. 2c). In the control, untreated emanators and HLC were used.

The FTPE were positioned in the experimental chambers 45 min before the experiment started to allow the release of active ingredients into the experimental space, and mosquitoes were transferred to the buffer chamber (corridor) of the SFS 1 h before the experiment began to allow for acclimatization. During the acclimatization process, mosquitoes remained free from transfluthrin exposure. After the acclimatization period, cages with approximately 25 mosquitoes each were positioned in the 4 corners of both compartments (approximately 100 mosquitoes per compartment/treatment). Mosquitoes were released at 07:00 h by a gentle pull of the strings connecting the releasing cages and the chair where volunteers were sitting. The experiment was conducted from 07:00 h to 10:00 h to reflect natural biting time for *Aedes* mosquitoes [25].

The volunteers conducted HLC, collecting mosquitoes that landed on the area between the ankle and the knee for 3 consecutive hours. All volunteers were males aged between 25–40. They were non-drinkers, non-smokers and were asked not to apply perfume or bathe using perfumed soap, before the experiments. During the experiment, volunteers wore shorts, covered shoes, and bug jackets to standardize the area available for mosquito landings. Mosquitoes were recaptured continuously for 50 min using a mouth aspirator. After 50 min the volunteers would take a break for 10 min, after which a new paper cup labeled with the time and date were used. Collected mosquitoes were transferred to the insectary for sorting. After the experiment, mosquitoes that were not collected during the HLC were recaptured using locally made Prokopack aspirators and killed by freezing to prepare the SFS for the next day’s experiment. A Tinytag® view 2 data logger (model TV-4500; Gemini data logger, Chichester, UK) was placed inside the SFS throughout the experiment to record temperature and relative humidity.

### Experiment to assess the longevity of the FTPE

To assess the longevity of FTPE protection, the devices were evaluated at 0, 3 and 6-months post-impregnation. The same set up as described previously (Fig. 2a) was followed. Between the evaluations, the emanators were “field aged” by storing the hessian strips in an outdoor environment under a tree in the shade to simulate aging on a verandah of a house, i.e. placed outdoors under ambient conditions, protected from direct sunlight and rain (Fig. 1e).

### Sample size

Sample size calculations were performed using simulation-based power analysis [26] in R statistical software version 3.02 (http://www.r-project.org) with a significance level of 0.05 for rejecting the null hypothesis. Data analysis for experimental data was conducted using generalized linear mixed models (GLMMs). Therefore, one thousand simulations of generalized linear mixed models approximating those that will be used to analyze project data were run using the same experimental design. The power to predict the difference in mosquito landings between control and treatment was estimated as the proportion of the 1000 simulated data sets in which the null hypothesis was rejected when the generalized linear mixed model was run. Parameters were set at 10% estimated variability between chambers, 10% variability between mosquito releases and 10% variability between volunteers. Simulations indicated that with an estimated 100 mosquitoes released per day and 60% recapture of released mosquitoes in the control, there was 94% power (95% CI: 92–96%) to detect a 50% reduction in mosquito landings in the treatment arm after 16 nights of experimentation. Furthermore, there was 70% power (95% CI: 68–72%) to detect a 15% difference between the treatments.

### Data analyses

Data were entered in Microsoft Excel 2010 and analyzed in Stata 13 (StataCorp, College Station, TX, USA). The data were analyzed to determine the efficacy of each intervention (push alone, pull alone and push-pull) to reduce the human landing rate compared to the control. The mean percentage recapture and confidence intervals were calculated for each intervention and control. From this daily protective efficacy was measured by comparing the human landing rate on a volunteer, with the intervention to the negative control using the following formula and the overall arithmetic mean PE and 95% confidence interval for the experiment was calculated:

$${\text{Protective efficacy }}\left( {\text{PE}} \right) \, = \, \left[ {\left( {{\text{C}}{-}{\text{T}}} \right)/{\text{C}}} \right] \, \times { 1}00\%$$

where C stands for the number of mosquitoes landing in the control and T is the number of mosquitoes landing in the treatment.

The effect of each intervention was determined by fitting a GLMM with a binomial distribution and logit function. The binomial distribution was chosen, as the dependent variable was the proportion of recaptured mosquitoes out of those released. Independent fixed effect categorical variables were treatment (intervention or control), compartment, volunteer, block (push, pull, push-pull) and an interaction term between treatment and block. Day was included as a random effect.

To determine the longevity of FTPE across 6 months after impregnation; a GLMM with a binomial distribution and logit function was also used. For this model, the dependent variable was again the proportion of recaptured mosquitoes. Independent fixed effect categorical variables were treatment (intervention or control), compartment, volunteer, month of testing (month 0, month 3, month 6) and an interaction term between treatment and months. This interaction was used to determine if the protective efficacy of FTPE changed between months. Day was included as a random effect.

During the evaluation, there was no significant association between humidity and temperature on the proportion of recaptured mosquitoes *P* = 0.705 and *P* = 0.203 for the humidity and temperature, respectively. Therefore, these variables were not included in the GLMM.

## Results

### Protective efficacy of the push-alone, pull-alone and push-pull

During the 16 experiment days, 439/1600 (27%) of released mosquitoes were captured in the presence of FTPE, 926/1600 (58%) in the presence of BGS and 349/1600 (22%) in the presence of push-pull, whereas in the control compartment, 951–1114/1600 (59–71%) of released mosquitoes were recaptured (Table 1). The average temperature during the experiments was 25.4 °C (21.0–26.0 °C) and the average relative humidity was 90% (68–100%). The full dataset is available in Additional file 1: Dataset S1.

**Table 1:** The percentage of mosquito landings, protective efficacy and the odds ratio for each intervention

Experiment	Treatment	Control	PE (95% CI)	OR (95% CI)	*P*-value
Push	439/1600 (27%)	1141/1600 (71%)	61.2% (52.2–69.9)	0.14 (0.12–0.16)	< 0.0001
Pull	926/1600 (58%)	951/1600 (59%)	2.1% (−2.9–7.2)	0.92 (0.81–1.08)	0.371
Push pull	349/1600 (22%)	999/1600 (62%)	64.5% (59.1–69.9)	0.16 (0.14–0.19)	< 0.0001

*Notes*: Numbers in the control and treatment groups refer to the total number of mosquitoes caught/released during each experiment and the percentage recaptured are in parentheses. Also shown are the estimates for PE and 95% CI. Finally, the OR and *P*-value derived from the GLMM model are presented

**Fig. 3:**
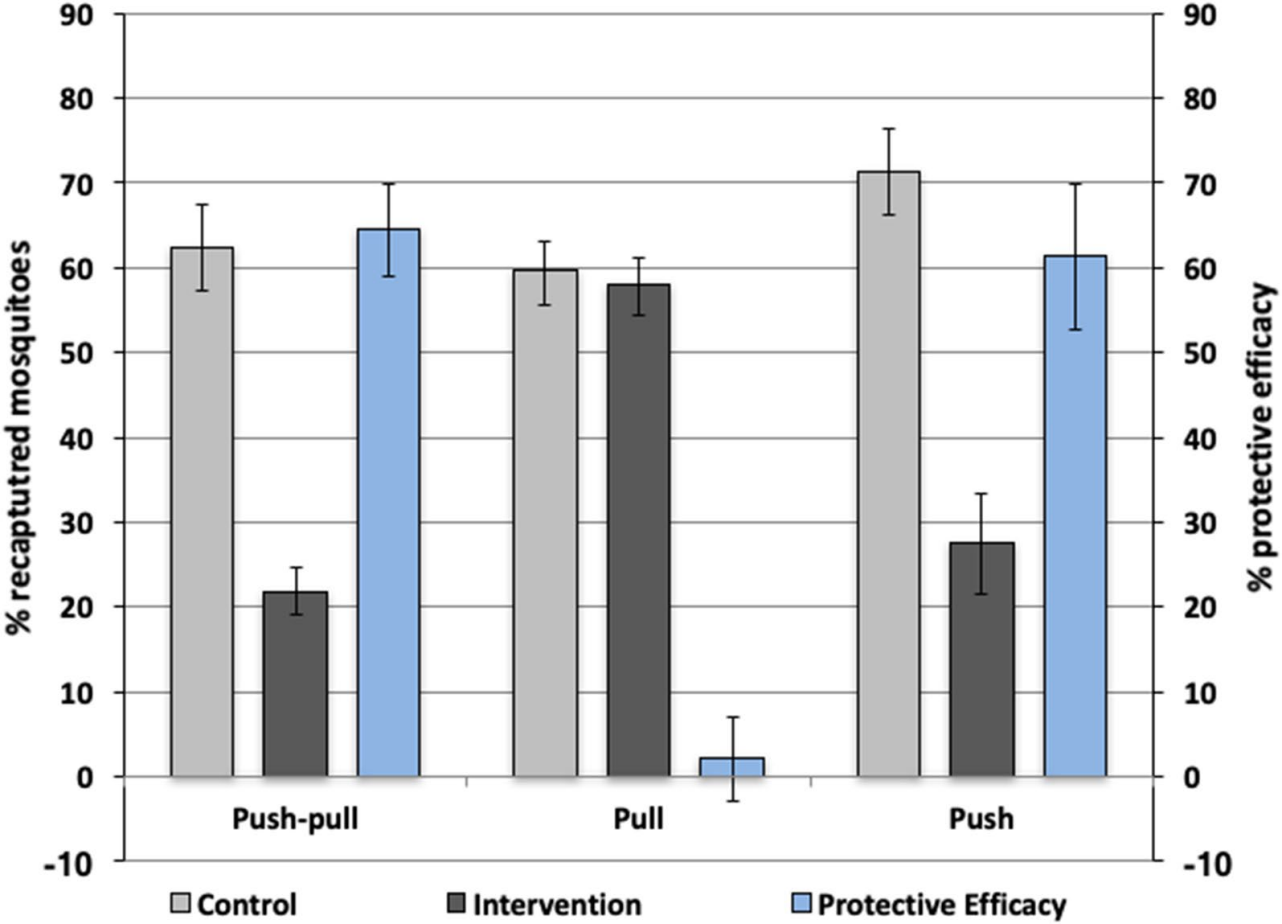
Percentage of recaptured mosquitoes and protective efficacy. The arithmetic mean percentage of mosquitoes recaptured by HLC in the presence of the BGS (pull), FTPE (push), spatial repellent emanator and odour-baited trap (push-pull) compared to the control. The secondary axis shows the % protective efficacy of each intervention. Error bars represent the 95% confidence intervals

The protective efficacy (PE) of FTPE against *Ae. aegypti* bites was 61.0% (95% CI: 52.2–69.9%; odds ratio (OR): 0.14, 95% CI: 0.12–0.16, *P* < 0.0001) (Fig. 3, Table 1). The BGS did not reduce *Ae. aegypti* landings on a human volunteer sitting 10 m away with PE of 2.1% (95% CI: −2.9–7.2%; OR: 0.92, 95% CI: 0.81–1.08, *P* = 0.371) (Fig. 3). The combination of FTPE and the BGS significantly reduced *Ae. aegypti* landings with PE of 64.5% (95% CI: 59.1–69.9%; OR: 0.16, 95% CI: 0.14–0.19, *P* < 0.0001) (Fig. 3, Table 1). The proportion of mosquitoes caught by BGS used alone 6.1% (95% CI: 5.1–6.1%) or in combination with FTPE 6.1% (95% CI: 5.0–7.3%) showed no significant difference (*P* = 0.34). This indicates that the push and pull components were not working synergistically, with a majority of protection provided by the push alone.

### Comparing the performance of the push-alone, pull-alone and push-pull

A significant interaction between type of intervention (push, pull or push-pull) and treatment in the model confirmed that the majority of protective efficacy in the push-pull was provided by the FTPE. The protective efficacy of push-alone, as described above, was significantly greater than pull-alone (*P* < 0.0001) while the protective efficacy of push-alone was not significantly different than the full push-pull system (*P* = 0.29). There was no significant difference in the compartment (*P* = 0.29) or volunteers (*P* > 0.05 for all volunteers) on the number of recaptured mosquitoes.

### Protective efficacy of the push-alone over six months

**Fig. 4:**
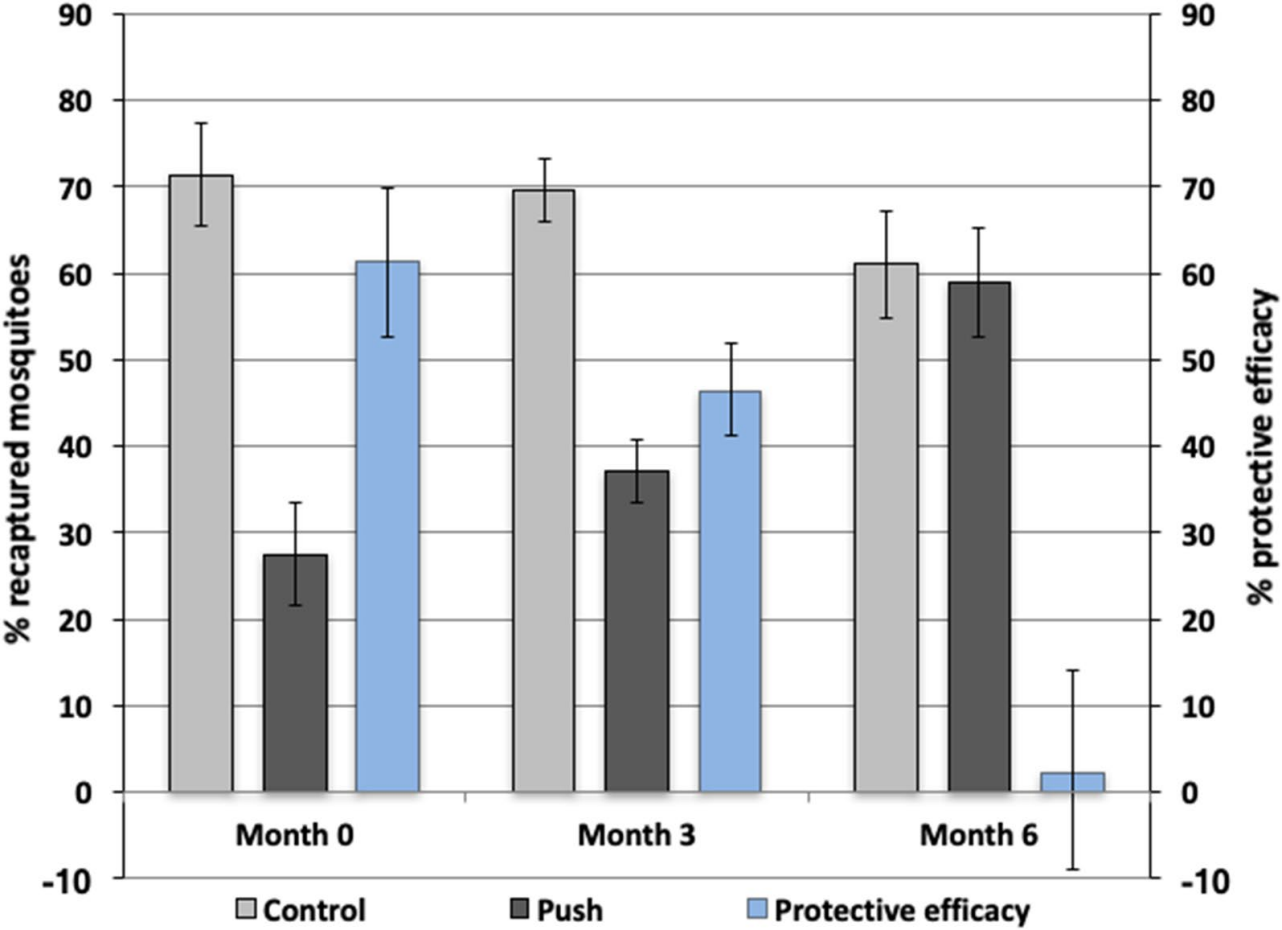
The duration of efficacy of the FTPE. The arithmetic mean percentage of mosquitoes recaptured by HLC in the compartment with FTPE compared to the control up to six months after treatment. The secondary axis represents the % protective efficacy of the push at each time point. Error bars represent the 95% confidence intervals

There was a significant interaction between month and treatment showing that the PE of the FTPE decreased over time (*P* < 0.0001). At 3 months after impregnation, the FTPE was still providing significant protection against *Ae. aegypti* with a PE of 46.4% (95% CI: 41.1–51.8%; OR: 0.26, 95% CI: 0.22–0.29, *P* < 0.0001). However, when the FTPE were tested at month 6 after impregnation, no significant protection was offered (OR: 0.91, 95% CI: 0.79–1.05, *P* = 0.22), with protective efficacy dropping to 2.2% (95% CI: −9.0–14.0) (Fig. 4).

## Discussion

The current vector control tools used against *Aedes* mosquitoes have several limitations, necessitating the development and testing of additional tools for proactive dengue prevention. This study demonstrated that while the push-pull system reduced human-vector contact of *Ae. aegypti* mosquitoes, the majority of protection was provided by the FTPE. The likely reason for this is the poor response of mosquitoes to the BGS in the presence of humans. Numerous previous studies on push-pull technologies have demonstrated the higher efficacy of the push than the pull [14, 27–29]. While the push-pull system may need further development, the success of the FTPE was encouraging and indicated their potential for the control of arboviral diseases.

The FTPE remained protective for three months, therefore, it is possible to meet high levels of coverage in an urban setting with just one application, reducing the difficulties of re-application and user non-compliance. These promising results indicate that FTPE could potentially be used to protect individuals in the peridomestic space longer than current personal protection methods [5]. This may be particularly useful during arboviral disease outbreaks that tend to coincide with the 3–5-month rainy season [30].

Our finding of FTPE efficacy is consistent with previous studies evaluating transfluthrin against *Anopheles arabiensis* and *Ae. aegypti* mosquitoes [31–33]. However, we may not generalize that these transfluthrin-treated passive emanators provide protection in all geographical locations. It is important to consider environmental factors and susceptibility status of the mosquitoes before implementing any control strategy. For example, in a windy environment, the active ingredient can be blown away, therefore reducing the concentration needed in the air to repel mosquitoes. Temperature can affect the vaporization of transfluthrin and thereby its concentration and protective efficacy. It has been reported that the optimal temperature for a transfluthrin-treated emanator to provide maximum protection ranges between 21–30 °C, with a reduction in protection specifically in lower temperatures [11]. This suggests that in geographical locations where the daytime ambient temperature is below 21 °C the efficacy of these emanators for prevention of *Ae. aegypti* bites may be impaired. However, these experiments were conducted within the temperature range of 20.9–25.5 °C, which is optimal for transfluthrin evaporation. Also, it was reported that resistance to transfluthrin could be selected for among laboratory *Ae. aegypti* which were less sensitive to transfluthrin afterward [34]. This implies that the efficacy of these emanators may be impaired in the area with confirmed pyrethroid resistant *Aedes* mosquitoes.

In this study, we have demonstrated that the BGS positioned 10 meters away did not significantly protect a person from mosquito bites. However, previous experiments have shown that the BGS used alone is an effective trap for sampling *Ae. aegypti* [17, 23, 35]. In this experiment, the BGS was placed near a human volunteer and they were the only “hosts” available. This demonstrated that the human cues were significantly more attractive to *Aedes* than the cues from the BGS. Preliminary work in the semi-field system indicated that the BGS caught many *Aedes* in the absence of the human volunteers, revealing that the efficacy of BGS is relative to the proximity and density of humans. This has also been observed in other studies with humans outcompeting traps at short range [12] and that whole human odor is optimally attractive to anthropophagic mosquitoes [36]. While the BGS did not provide personal protection by reducing human-vector contact as the removal trap, it could still provide some level of community protection if used on a larger scale, although other traps such as the autocidal gravid trap may be more feasible for removal trapping [37] as they do not require carbon dioxide.

The number of mosquitoes successfully caught by the BGS during the push-pull or the pull only configuration was the same. While this showed that transfluthrin did not actively push mosquitoes into the trap it also indicated that transfluthrin exposure outdoors does not inhibit mosquitoes entering the BGS. This is contrary to Salazar et al. [38] who reported that exposing mosquitoes to transfluthrin significantly lowered trap catches. However, trap catches were not affected if the mosquitoes were allowed to recover for 12 h before BGS trap evaluation [38]. This suggests that the mode of action of transfluthrin is dose- and distance-dependent and repellency is reversible (i.e. mosquitoes are disarmed for a period). Use of a higher dose could be further optimized to increase the kill of mosquitoes or cause a prolonged state where mosquitoes are disarmed and unable to find a host. This could prevent diversion of repelled mosquitoes from repellent users to non-users in a community [39].

We have shown that FTPE remain protective for three months following impregnation. This is a relatively short duration compared to the previous studies conducted against malaria vectors demonstrating that transfluthrin-treated strips remain protective for up to six months against *Anopheles* mosquitoes [11, 15]. A possible explanation for these differences could be due to the variation in transfluthrin dosage, mosquito species and the distance from the emanator where HLC was performed. *Aedes aegypti* is highly anthropophilic and requires higher doses of topical repellents to be repelled than other species with more diverse host preferences such as *An. arabiensis* [40]. In the present study, 10.5 g of transfluthrin (5.25 g on each of two FTPE) was used against *Ae. aegypti* and HLC conducted three meters from the emanators, whereas in the study by Ogoma et al. [11, 15] the volunteer sat one meter from a strip enclosing them on all four sides at an application of 15.1 g transfluthrin against *An. arabiensis* mosquitoes. Ogoma et al. showed that hessian treated with 1.51 g of transfluthrin resulted in air concentration > 1000 times lower than the maximum acceptable concentration for long term inhalation exposure of a human being as defined by the regulatory authorities of the European Union (EU) [11]. In general, the efficacy of the emanators in both studies decreases over time as the result of the loss of transfluthrin due to evaporation. To ensure long-term efficacy of the FTPE, a thicker layer of the hessian strips could be used or transfluthrin doses increased, provided they remain within the margin of safety for chronic inhalation exposure [41]. The FTPE is a simple proof of concept prototype and further work is required to develop a product for use as a public health intervention, including standardizing the release rate of the transfluthrin through standardization of the material upon which the transfluthrin is applied, and improvement of the delivery unit to ensure it is cost-effective and tamper-proof. This may also include the application of UV protection to the transfluthrin-treated material to prolong its efficacy outdoors [42].

The use of FTPE improves user compliance, as they are portable and easy to use which facilitates round the clock protection at the desired location in the peridomestic area. The replacement of FTPE after every three months avoids the problems associated with personal topical repellents that require daily application, but tend to be applied only when people notice mosquito bites [43, 44], resulting in lack of public health benefit [45]. As the device potentially provides protection to multiple people without the need for personal reapplication, it is likely a convenient approach to bite prevention outside of sleeping hours and to be more acceptable among community members for protection of the whole family [46]. Therefore, the FTPE are suitable for targeted distribution among high-risk populations such as those reported to harbor *Aedes* breeding sites during the outbreak [47]. Finally, the FTPE is a passive device that does not produce smoke (like mosquito coils), which improves user acceptance and reduces potential exposure to organic pollutants.

Dengue tends to be focal in transmission with *Aedes* spp. commonly having a short flight range, although there are exceptions [48]. Therefore, transmission is primarily mediated between locations by movement of infected individuals [49]. These devices are portable and may be deployed anywhere; they could be very useful if provided to those households with confirmed dengue, deployed in entrance points (port and airport) where travelers are coming in from other countries, or in places where new cases are suspected or an outbreak is reported, such as markets [1]. The high mosquito toxicity of transfluthrin is an important feature of this tool, as it has the potential to kill a substantial proportion of mosquitoes that encounter the insecticide and reduce vector densities and vectoral capacity. Further work into the impact of such devices on the resistant *Ae. aegypti* and mortality of free-flying mosquitoes is recommended.

There were two limitations of this study, including the use of laboratory-reared mosquitoes that may exhibit different behaviors to wild mosquitoes. Also, the data for the longevity experiment were collected at 0, 3 and 6 months only. Whereas significant protective efficacy (44%) was observed up to three months after impregnation the FTPE. With this experimental design we missed the exact time point (between 3 and 6 months) when the FTPE stopped providing significant protection. We recommend that future studies conducting the same kind of experiment need to conduct weekly or monthly evaluations in order to provide a more precise estimate of efficacy over time, especially when testing the label claims of long-lasting spatial repellent products. Moreover, we only evaluated one distance setup for use of the “push-pull” in the SFS. In the field, the positioning of this system will vary due to the local building layout resulting in varying protection levels. We recommend that further studies on push-pull should (i) focus on improving the attraction of pull components, since they need to outcompete humans, and (ii) explore optimal positioning of components to determine the effective distance at which the push and pull could work synergistically.

## Conclusions

In this study, we have demonstrated that FTPE has potential to reduce bites from *Ae. aegypti* mosquitoes for up to three months. Using a combination of passively emanated transfluthrin and BGS as a push-pull did not provide any additional protection, with the majority of the protection originating from the push component. Additional work is needed in the field and through mathematical modeling to determine if the number of mosquitoes caught in the BGS would provide additional community protection when added to the FTPE. The FTPE are portable and easy to use which facilitates round the clock protection at the desired location in the peridomestic area for much longer than most currently available personal protection methods.

## Supplementary information

**Additional file 1: Dataset S1.** The dataset from the semi-field evaluation of the push pull for the control of outdoor biting mosquitoes that support the conclusions of this article.

## Data Availability

All data generated or analysed during this study are included in this published article and its additional file.

## Abbreviations

FTPE: Freestanding transfluthrin passive emanators
CI: Confidence interval
DEET: N,N-diethyl-meta-toluamide
BGS: Biogents sentinel trap
BGL: Biogents lure
EIR: Entomological inoculation rate
EC: Emulsifiable concentrate
SFS: Semi field system
HLC: Human landing catches
GLMM: Generalized linear mixed model
PE: Protective efficacy
UV: Ultraviolet
IHI: Ifakara Health Institute
IRB: Institute Review Board
NIMR: National Medical Research Institute
WHO: World Health Organization
